# Transcranial alternating current stimulation improves cognitive functions in healthy subjects through modifying frontoparietal and dorsal attention networks based on personalized individual theta frequency analysis

**DOI:** 10.3389/fnbeh.2026.1821101

**Published:** 2026-06-12

**Authors:** Burak Yulug, Ali Yalcinkaya, Ayse Karakuş, Shair Shah Safa, Dila Sayman, Alexandra Berekelia, Seyda Cankaya, Ceyhun Sayman, Sevilay Ayyildiz, Behçet Ayyildiz, Mehmet Ozansoy, Halil Aziz Velioglu, Lutfu Hanoglu, Adil Mardinoglu

**Affiliations:** 1 Department of Neurology and Neuroscience, Alanya Alaaddin Keykubat University, Antalya, Türkiye; 2 Department of Neurology, Istanbul Medipol University, Istanbul, Türkiye; 3 School of Medicine and Health, TUM-NIC Neuroimaging Center, Technical University of Munich, Munich, Germany; 4 Department of Physiology, International School of Medicine, Istanbul Medipol University, Istanbul, Türkiye; 5 Department of Neuroradiology, Center for Psychiatric Neuroscience, Feinstein Institute for Medical Research, Manhasset, NY, United States; 6 Functional Imaging and Cognitive-Affective Neuroscience Lab (fINCAN), Health Sciences and Technology Research Institute (SABITA), Istanbul Medipol University, Istanbul, Türkiye; 7 Science for Life Laboratory, KTH-Royal Institute of Technology, Stockholm, Sweden

**Keywords:** cognitive function, dorsal attention network, EEG, fMRI, frontoparietal network, memory enhancement, transcranial alternating current stimulation (tACS)

## Abstract

**Introduction:**

Transcranial alternating current stimulation (tACS) has emerged as a promising tool to modulate cognitive functions by entraining endogenous neural oscillations. This study investigated the behavioral and neurophysiological after-effects of theta-frequency tACS individualized to each participant’s intrinsic theta frequency (ITF).

**Methods:**

Twenty-two healthy participants were randomly assigned to either a real stimulation group (tACS group) or a sham group. Cognitive assessments and resting-state EEG/fMRI data were collected pre- and post-stimulation.

**Results:**

Participants in the real tACS group showed significant post-stimulation improvements in short-term memory, verbal fluency and category fluency scores compared to the sham group. Functional connectivity analyses revealed increased activity in the dorsal attention and frontoparietal networks only in the stimulation group, suggesting network-specific modulation

**Discussion:**

These effects align with the theta-gamma coupling theory and provide evidence for long-lasting neuroplastic changes following personalized tACS. This study contributes to our understanding of brain stimulation and supports the use of tACS for cognitive enhancement in healthy individuals.

## Introduction

Transcranial alternating current stimulation (tACS) has been employed to modulate and entrain endogenous brain oscillations, thus allowing the causal investigation of hypothesized roles of oscillations in human cognition (Bland and Sale, 2019). tACS delivers low-intensity electrical currents between two electrodes, alternating in polarity at predetermined frequencies associated with targeted cognitive processes. Since a slow oscillating theta can accommodate more cycles of fast gamma, previous studies have investigated whether tACS can be employed to increase memory capacity by reducing the frequency of theta (Aktürk et al., 2022). Memory processes are fundamental to human cognition, being closely related to other core functions such as attention and executive control. It is therefore not surprising to observe the interaction of theta oscillatory characteristics described above with other frequency bands associated with high-level cognitive functions (Polanía et al., 2012). In that context, theta-gamma coupling appears to represent an ideal mechanism for linking representations or operations from different neurocognitive sources into a cohesive mental representation (Polanía et al., 2012). Lisman and Idiart (1995), for instance, proposed that the number of gamma cycles fitting into one theta cycle determines the limits of memory capacity. In other words, a longer theta cycle implies a higher memory capacity by accommodating more gamma cycles. Several correlational studies have provided evidence supporting this proposed relationship between memory performance and theta-gamma cross-frequency coupling (Jaušovec and Jaušovec, 2014; Wolinski et al., 2018; Sreeraj et al., 2019; Aktürk et al., 2022; Manippa et al., 2024).

The majority of these studies have employed tACS to entrain brain oscillations and enhance or modulate cognitive functions during its application (i.e., online effects) (Jaušovec and Jaušovec, 2014; Wolinski et al., 2018; Sreeraj et al., 2019). Although these effects are intriguing and promising, online tACS effects may be potentially confounded by sensory entrainment effects (indirect entrainment) resulting from the sensation of the tACS application on the skin. In contrast, successfully demonstrating modulatory effects beyond the stimulation period (after-effects) would not only be less confounded by these sensory stimulation side-effects but would also be crucial for acquiring new insights into the oscillatory processes underlying potential tACS-induced changes. Furthermore, offline tACS memory effects would also pave the way for greater clinical and applied relevance in using tACS for cognitive rehabilitation and/or neurocognitive enhancement (Vosskuhl et al., 2015). Most offline effects on neural oscillations, on the other hand, can be observed over considerably longer time periods. However, although there are some promising studies on the after-effects of “theta” tACS on both brain activity and behavior, the majority did not provide direct evidence of the behavioral after-effect of theta tACS (Jaušovec and Jaušovec, 2014; Vosskuhl et al., 2015; Sreeraj et al., 2019; Aktürk et al., 2022).

These findings regarding the after-effects of tACS are primarily discussed in the context of the mechanism of “timing-dependent plasticity” (Polanía et al., 2012). However, the theta-frequency after-effects on the EEG correlates of tACS-induced behavioral changes related to spatial correlates are not as well-established in the existing literature (Aktürk et al., 2022). Exploring the behavioral and physiological after-effects of theta tACS, as presented here, is therefore of crucial importance for acquiring an improved understanding of the potential plasticity-related changes of tACS concerning spatial resolution that extend beyond the transient time-dependent entrainment effects. Additionally, beyond its established pro-cognitive effects derived from various coupling protocols in conditions associated with cognitive impairment, such as Alzheimer’s disease, Parkinson’s disease, multiple sclerosis, and psychiatric challenges such as schizophrenia (Llufriu et al., 2017; Del Felice et al., 2019; Reinhart and Nguyen, 2019; Sreeraj et al., 2019; Traikapi and Konstantinou, 2021; Lee et al., 2022), tACS stimulation has also demonstrated benefits in healthy individuals (Sun et al., 2022; Manippa et al., 2024). For instance, Meiron and Lavidor (2014) demonstrated that tACS stimulation over the bilateral DLPFC region improved participants’ online working memory functions. This was corroborated by another novel study indicating that tACS enhanced short-term memory functions by altering individual theta frequency (ITF) in 35 healthy individuals. One intriguing aspect of that study was that the authors reduced ITF using tACS while simultaneously monitoring stimulation with EEG (Meiron and Lavidor, 2014). Ultimately, the authors concluded that lowering ITF resulted in improved short-term memory in healthy individuals. Sun et al. (2022) administered 10 Hz tACS to the prefrontal cortex of 18 healthy individuals, leading to significant enhancements in verbal fluency, a key cognitive subdomain. Similarly, Nissim et al. (2023) reported improved working memory functions following tACS stimulation in both Alzheimer’s disease patients and healthy individuals. There is increasing evidence that gamma-tACS enhances cognitive functions. A notable example of this is a recent study by Manippa et al. (2024), which examined the effects of temporal gamma-tACS and auditory stimulation on verbal memory in healthy adults. The findings suggest that this combined neuromodulation approach can enhance verbal memory, underscoring its potential use for cognitive enhancement strategies. Additionally, Polanía et al. (2012) investigated theta-synchronized tACS in healthy individuals performing a delayed letter discrimination task while receiving tACS at 6 Hz over the left prefrontal and parietal cortices, in either a 0° (“synchronized” condition) or 180° (“desynchronized” condition) phase. The results indicated that externally induced frontoparietal theta synchronization significantly improved visual memory matching reaction times compared to sham stimulation, while theta desynchronization exhibited a reverse pattern and impaired cognitive performance. This suggests that theta phase synchronization between distant cortical areas plays a causal role in cognitive performance in healthy humans and that tACS is a suitable method for artificially synchronizing or disrupting behaviorally relevant brain rhythms (Polanía et al., 2012).

As previously discussed, several studies in the literature have suggested that slowing an individual’s theta peak frequency may enhance cognitive performance. This effect has been interpreted within the framework of the theta–gamma coding hypothesis, which proposes that reducing theta frequency may allow a greater number of gamma cycles to be nested within a single theta cycle, thereby increasing the brain’s information-processing and working memory capacity. Accordingly, theta tACS administered at frequencies lower than the individual theta peak may facilitate cognitive functions by modulating theta–gamma coupling dynamics. In a study conducted by Ociepka et al. (2025), participants’ individual theta frequencies were first determined, after which tACS was applied at a frequency 1.5 Hz below each participant’s individual theta peak frequency. The authors reported that working memory performance was significantly improved in the active stimulation group compared with the sham group (Ociepka et al., 2025). Similarly, in a study by Vosskuhl et al. (2015), tACS was administered at a frequency lower than the participants’ individual theta peak frequency, and the real tACS group demonstrated improved short-term memory performance compared with the sham group.

There is also a limited number of exceptional studies combining fMRI with tACS and cognition in healthy individuals. For instance, Seo et al. (2025) collected task-based (working memory task) functional magnetic resonance images from healthy participants and applied tACS to the DLPFC and bilateral posterior parietal cortex. Their findings demonstrated activation of the hippocampus and precuneus, key regions for working memory processing (Seo et al., 2025). Although it did not consider the pro-cognitive effects of tACS. Another study by Ma et al., (2025) compared real tACS with sham stimulation using resting state EEG and fMRI. Their findings revealed that alpha power temporal fluctuations in EEG were coupled with default mode network (DMN) connectivity. However, a more recent study indicated that theta tACS on the left parietal cortex enhances intelligence performance (as assessed by Raven’s test) while reducing activation in the left inferior parietal cortex (Neubauer et al., 2017).

The current study assessed the potential after-effects of a tACS protocol aiming to “slow down” the individual theta frequency (ITF), with the aim of enhancing memory performance capacity beyond the period of stimulation. Furthermore, we evaluated the after-effects on resting-state fMRI, focusing on theta-band oscillations. For that purpose, we administered tACS at 1 Hz slower than the individual theta frequency (ITF-1) of each participant to experimentally reduce the theta frequency and thus improve memory performance. Using a between-subjects design, participants were randomly assigned to two stimulation conditions: Real tACS stimulation at ITF-1 (for slowing down ITF, changing the frequency but with no or less entrainment), and sham stimulation. TACS was applied to the left dorsolateral prefrontal cortex network, which is part of the neural networks underlying memory and learning functions (Cabeza and Nyberg, 2000).

Based on our *a priori* hypothesis-driven main research question, tACS-related cognitive after-effects were anticipated, particularly in specific executive and memory-related tasks and for the real stimulation [which is individual theta frequency minus 1 (ITF-1)] group, due to the induced “theta slowing” compared to sham. Since our aim was to study the after-effects of tACS, stimulation was applied at rest for 20 min, and memory and learning task results, along with fMRI measurements, were obtained both before and after the tACS interventions.

## Materials and methods

**Figure 1:**
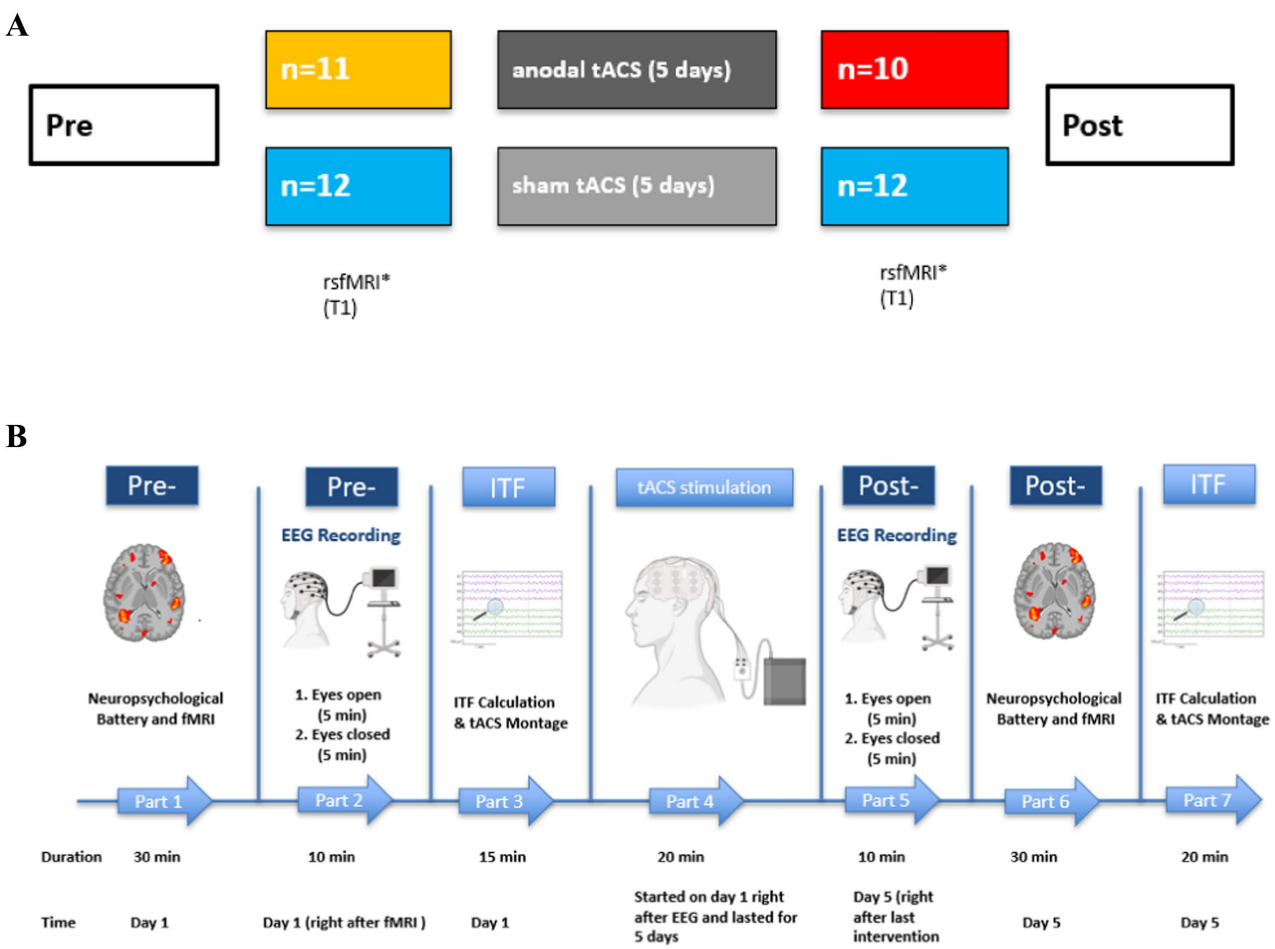
Study design illustrating stimulation protocol and imaging timeline. (**A**) Participants were randomly assigned to receive either active transcranial alternating current stimulation (tACS; *n* = 11) or sham stimulation (*n* = 12) for five consecutive days. 11 participants were assigned to the real stimulation group and 12 to the sham stimulation group randomly, resulting in a total of 23 participants. But one participant from the real tACS group withdrew from the study before completing the intervention. 10 participants from the real tACS group and 12 participants from the sham group (totally 22 participants) completed the intervention and enrolled in the analysis of the study. Resting-state functional MRI (rsfMRI) scans were acquired at baseline (Pre) and after the intervention period (Post) to assess neural connectivity changes associated with the stimulation. (**B**) Schematic overview of the study design. Neuropsychological assessment, resting-state fMRI, EEG recording, and individual theta frequency (ITF) analysis were performed both before and after the 5-day tACS intervention. tACS, transcranial alternating current stimulation; rsfMRI, resting state functional magnetic resonance imaging.

In this study we aimed to evaluate the effect of tACS on cognition, memory, executive function, attention and language skills in healthy individuals. The study was designed as a single-blind, randomized controlled study. 22 healthy participants (10 of them received real stimulation, 12 of them received sham stimulation) recruited to the study based on inclusion and exclusion criteria. Inclusion criteria were defined as: providing informed consent; Being literate and 20–35 years of age; having no psychiatric or neurological disorder; not using any medication that affects cognition, memory and affective state. Exclusion criteria were defined as: Illiteracy or educated to lower than primary school level; presence of an existing and/or previous neurological disease, psychiatric disease or head trauma, and/or irreversible hearing or vision problems or other medical illness (e.g., depression, personality disorders, psychotic disorders, autism etc.); presence of medical issues that prevent the application of electroencephalography or tACS. Participants’ depressive status has been evaluated with Hamilton Depression Rating Scale (HDRS). Neurocognitive assessments were conducted to evaluate multiple cognitive domains before and after tACS stimulation. Global cognitive function was assessed using the Mini-Mental State Examination (MMSE) and Montreal Cognitive Assessment (MoCA). Attention and executive functions were evaluated using the Stroop Test (Stroop Tests 1–3) and the Trail Making Test (Parts A and B). Memory was assessed using short-term memory and recall measures, as well as the Wechsler Memory Scale (Visual Memory subtest). Language functions were evaluated using category fluency and verbal fluency tests. And depression level evaluations also were conducted before and after tACS stimulation. rsfMRI also done before and after tACS and sham stimulation (Figures 1A,B). We also conducted EEG before tACS stimulation to determine the individual’s theta frequency. Personalized tACS stimulation (Individual theta frequency minus 1 (ITF-1)) was administered to the dorsolateral prefrontal cortex (DLPFC) area, utilizing individual theta frequency (ITF) determined with MATLAB. The 20-min sessions, spanning 5 days, employed a 2-mA sinusoidal oscillating current at minus one ITF (4–8 Hz) to enhance cognitive functions. Post-stimulation, participants underwent the same battery of cognitive tests and EEG scans.

### tACS

Participants were seated in a comfortable chair in a quiet room and instructed to remain rested with their eyes open during the stimulation session. The stimulation session was 20 min and personalized (Individual theta frequency minus 1 (ITF-1)) in both real tACS and sham stimulation groups. To perform the tACS, we used NeuroMars (MARS-EV01) stimulator. The direct current was delivered via 5*5 cm-wide medical level conductive pads. As previously mentioned, numerous studies in literature have applied tACS over the dorsolateral prefrontal cortex (DLPFC) in order to enhance cognitive performance (Meiron and Lavidor, 2014; Seo et al., 2025). Therefore, based on this existing evidence, we also selected the DLPFC as the stimulation target in our study. The stimulation electrode was placed over the left dorsolateral prefrontal cortex (DLPFC), corresponding to the F3 position, and the return electrode was positioned over CP6 (temporoparietal junction), based on the international 10–20 EEG system. The current density was 0.06 mA/cm2 from each electrode, with a total density of 0.054/cm2. The stimulation was delivered for 20 min per session, once daily, over five consecutive days. First intervention session started right after fMRI and EEG analyses and after the last (fifth) tACS intervention the post-intervention fMRI and EEG analyses conducted immediately. For the sham protocol, the stimulation was delivered at a very low current intensity, enough to cause slight tingling for 15 s and the sham session was also 20 min. The stimulation protocol has been arranged based on prior research in the field (Aktürk et al., 2022). The 20-min sessions, conducted over five consecutive days, involved sinusoidal oscillatory stimulation at an individual theta frequency minus 1 Hz (4–8 Hz), delivered at 2 mA in the real tACS condition and at 0.2 mA in the sham condition to induce mild tingling sensations.

Electrode locations were identified using standardized anatomical landmarks (nasion, inion, and preauricular points), which are widely used to ensure reproducible positioning across participants. To minimize electrode drift during stimulation, electrodes were secured using elastic straps and conductive gel, and participants were instructed to remain as still as possible throughout the session. Additionally, all stimulation sessions were conducted by the same trained researcher, which reduced variability related to inter-rater differences.

### Electroencephalography

Electroencephalography is a non-invasive imaging tool that evaluates changes in electrical activity in the brain. It is applied to the scalp via metal electrodes and a conductive media instrument. In this study, electroencephalography was used to explore electrophysiological alterations that occurred as a result of transcranial alternating current stimulation. EEG signals was recorded using the Neuroscan Synamps 2 system with a 64-channel (Ag/AgCl) electrode cap positioned according to the international 10–20 system. The active electrode was placed on left DLPFC/F3 and the return electrode was placed on temporoparietal junction/CP6. The electrode impedance values were monitored as a reference value of <50 kΩ. During the task, the participants were instructed to remain in an alert state. Resting-state EEG was recorded for 10 min with the patient’s eyes closed and open (5 min each). EEG data was obtained at baseline before the intervention, and at the end of the tACS stimulation.

### Primary outcome

The primary outcome of this study was to evaluate whether transcranial alternating current stimulation (tACS) led to a significant improvement in neurocognitive test scores (clinical improvement) from baseline to post-stimulation in the real stimulation group compared to the sham group.

### Secondary outcome

The secondary outcome involved examining functional connectivity alterations between groups following tACS and sham interventions and its correlation with neurocognitive test scores to further assess the impact of tACS on cognitive performance.

### Sample size

A G*Power analysis based on the findings of Kasten et al. (2016) was conducted to estimate the required sample size (Kasten et al., 2016). The results indicated that a total sample size of 16 (8 participants for each group) would provide sufficient statistical power (1 − *β* = 0.80). Accordingly, 11 participants were assigned to the real stimulation group and 12 to the sham stimulation group randomly, resulting in a total of 23 participants. But one participant from the real tACS group withdrew from the study before completing the intervention. 10 participants from the real tACS group and 12 participants from the sham group (totally 22 participants) completed the intervention and enrolled in the analysis of the study (Figures 1A,B).

### Data collection

Participant recruitment was conducted through an open call announced on the Alanya Alaaddin Keykubat University’s student bulletin board. Individuals who expressed interest in participating were directed to the neurology department of the affiliated hospital, where they were provided with detailed information about the study and signed informed consent forms. Participants were subsequently screened based on predefined inclusion and exclusion criteria. Demographic data, including age, gender, and educational background, were collected.

Neuropsychological assessments were administered by a qualified neuropsychologist. Electroencephalography (EEG) recordings were obtained at baseline and following transcranial alternating current stimulation (tACS). After the stimulation phase, neuropsychological assessments were repeated to evaluate potential changes.

### Data monitoring

The study design was applied in line with the Declaration of Helsinki and approved by the Clinical Research Ethics Committee of Istanbul Medipol University (Ethical Number: E-10840098-772.02-735). The imaging data was obtained in accordance with these guidelines, the same imaging devices was used all throughout the study, and the neuroassement was administered by the same neuropsychologists to all the participants.

### EEG preprocessing

EEG analysis was performed using the Brain Vision Analyzer 2.1 Software (Brain products, Munich, Germany). EEG recordings was analyzed total 19 electrodes (F3, F4, C3, C4, P3, P4, O1, O2, F7, F8, T1, T2, T3, T4, T5, T6, Fz, Cz, Pz) For the preprocessing EEG analyses, band-pass filter (IIR) of 0.1–60 Hz, with a 50 Hz notch was applied. The following procedure was used to remove artifacts (muscle and movement artifacts, eye movement etc.) from the EEG data: Firstly, raw data recordings were split into two primary segments, namely eyes open and eyes closed conditions, and assessed independently (for real and shame groups) during spontaneous EEG analyses. Then, data divided equal sized as 1 s epochs were used to separate the data for both eyes open and closed conditions. After this segmentation, artifact rejection was applied to remove eye movements, muscle movements and other artifacts from the recording during the offline.

### Spontaneous EEG with power spectrum analysis—ITF analysis

For the ITF calculation, raw data were initially imported into MATLAB using the EEGLAB toolbox (in ASCII format). Subsequently, frequency and amplitude filtering procedures were applied, and artifacts were removed. Finally, power spectral density and ITF values were computed through custom scripts. The ITF was defined as the frequency showing the maximum power within the 4–8 Hz theta range. Power spectral density (PSD) values were obtained using Fast Fourier Transform (FFT) analysis, and the frequency corresponding to the highest spectral power within the theta band was identified as the ITF for each participant. After artifacts rejection process, power spectrum analysis was used during spontaneous EEG recording. It measures the magnitude (or power) of waves. This performed by Fast Fourier Transform (FFT) (with 10% Hanning window and power was μV^2^). After each epoch’s FFT was obtained, FFT power values’ average of the epochs was obtained. Display range was between 0.5-48 Hz. The grand average of each electrode’s power spectrum was measured for the following frequency bands: delta (0.5-3.5 Hz), theta (4-7 Hz), alpha (8-13 Hz), beta (15-28 Hz), and gamma (28-48 Hz). According to the grand average of FFTs, major difference displayed in alpha frequencies band. Therefore, only alpha frequencies band’s power spectrum was measured. Each electrode’s maximum alpha values were obtained and utilized in statistical analysis. EEG recordings were made with the Nihon Kohden 1200 K device using a bipolar banana mount. Recordings were taken for 5 minutes with eyes closed and 5 minutes with eyes open, for a total of 10 minutes. The room where the recordings were taken was a quiet, dark, isolated room, and the patients stopped consuming caffeine up to 4 hours before the recording. EEG data was saved and exported as ASCII format. After loading the EEG data, the individual theta frequency and power spectral density were calculated using the EEGlab extension of the MATLAB programme and the following steps were performed in the order of: Firstly, the montage was converted from bipolar to monopolar(% Change montage from bipolar to monopolar), then filtering was done and the band-pass for theta frequency was set to 4-8 Hz.[% Step 1: Filtering (4-8 Hz bandpass filter)]. After filtering the record was divided into epochs and baseline correction process was performed(EEG = pop_rmbase(EEG, [baseline_start*1000 baseline_end*1000]). Then power spectral density (PSD) was calculated(% Calculate Power Spectral Density), after defining theta frequency, ITF was calculated.[(%Identify the Theta Band:theta_band_idx = find(freq >= 4 & freq <= 8);]theta_power = mean(PSD(theta_band_idx,:), 1)].

### Function MRI analysis

#### MRI data acquisition

Structural and resting-state fMRI was conducted using a 1.5 Tesla Signa Explorer MR device (General Electric Company, USA) at Alanya Alaaddin Keykubat University. Each T1-weighted structural scan consisted of 190 slices (TR/TE: 8.1/3.7), FOV 256 × 256 × 190 mm (FHxAPxRL), and a voxel size of 1 × 1 × 1 mm. Eyes-open resting state fMRI scan recordings were collected using an echo-planar imaging sequence (EPI). The scanning process lasted approximately 12 min, and 300 volumes were recorded with the following parameters: TR 2230 ms, TE 30 ms, FOV 240 × 240 × 140 mm (RLxAPxFH), voxel size 3 × 3 × 4 mm, flip angle 77^0^, and slice number 35. Before scanning, all participants were instructed to keep their eyes closed, relax and move as little as possible, to empty their minds, and not to fall asleep during the procedure.

#### Quality check

All participants’ MRI images were carefully reviewed by two researchers to verify their quality. It was confirmed that the anatomical and functional MRI images for all participants met the necessary standards for preprocessing. Following this, another round of assessment was performed. In this phase, the segmentation of T1-weighted anatomical images was checked, specifically focusing on the gray matter, white matter, and cerebrospinal fluid segmentation. The normalization of both functional and anatomical images to the MNI standard template was also visually inspected. Additionally, the images from the Freesurfer recon-all procedure were corrected through troubleshooting. The troubleshooting process involves removing any dura mater that may be present in the gray matter, resolving any confusion between white and gray matter, correcting topological defects in the white matter, addressing failures in the skull stripping procedure, and ensuring proper separation of white and gray matter by adding control points to regions misclassified as gray matter.

For functional images, several parameters were evaluated, including the number of valid and invalid scans, maximum and average motion, as well as maximum and average global signal changes. Participants were excluded from the study if 25% or more of their scans were deemed invalid, or if they exhibited extreme values in maximum/average motion or maximum/average global signal changes. Extreme values were defined as those that exceeded Q3 + 3 IQR or fell below Q1–3 IQR, where Q1 and Q3 represent the first and third quartiles of the measure’s distribution, respectively, and the interquartile range (IQR) is the difference between Q3 and Q1 (Morfini et al., 2023).

#### Analysis of resting-state functional connectivity (rsFC)

Analyses of fMRI data were performed using CONN (Whitfield-Gabrieli and Nieto-Castanon, 2012) (RRID:SCR_009550) release 21.a (Nieto-Castanon and Whitfield-Gabrieli, 2021) and SPM (Penny et al., 2007) (RRID:SCR_007037) release 12.7771.

##### Pre-processing

Functional and anatomical data were pre-processed using a modular pre-processing pipeline (Nieto-Castanon, 2020b,c) including realignment with correction of susceptibility distortion interactions, slice timing correction, outlier detection, direct segmentation and MNI-space normalization, and smoothing. Functional data were realigned using SPM realign & unwarp procedure (Andersson et al., 2001), where all scans were coregistered to a reference image (first scan of the first session) using a least squares approach and a 6-parameter (rigid body) transformation (Friston et al., 1995), and resampled using b-spline interpolation to correct for motion and magnetic susceptibility interactions. Temporal misalignment between different slices of the functional data (acquired in ascending order) was corrected following SPM slice-timing correction (STC) procedure (Henson, 1999; Sladky et al., 2011), using sinc temporal interpolation to resample each slice BOLD time series to a common mid-acquisition time. Potential outlier scans were identified using ART (Whitfield-Gabrieli and Nieto-Castanon, 2012) as acquisitions with framewise displacement above 0.9 mm or global BOLD signal changes above 5 standard deviations (Power et al., 2014; Nieto-Castanon, 2022), and a reference BOLD image was computed for each subject by averaging all scans excluding outliers. Functional and anatomical data were normalized into standard MNI space, segmented into grey matter, white matter, and CSF tissue classes, and resampled to 2 mm isotropic voxels following a direct normalization procedure (Nieto-Castanon, 2022) sing SPM unified segmentation and normalization algorithm (Ashburner and Friston, 2005; Ashburner, 2007) with the default IXI-549 tissue probability map template. Last, functional data were smoothed using spatial convolution with a Gaussian kernel of 6 mm full-width half maximum (FWHM).

##### Denoising

In addition, functional data were denoised using a standard denoising pipeline (Nieto-Castanon, 2020b,c) including the regression of potential confounding effects characterized by white matter time-series (5 CompCor noise components), CSF time-series (5 CompCor noise components), motion parameters and their first order derivatives (12 factors) (Friston et al., 1996), outlier scans (below 20 factors) (Power et al., 2014), session effects and their first order derivatives (2 factors), and linear trends (2 factors) within each functional run, followed by bandpass frequency filtering of the BOLD time-series (Hallquist et al., 2013) between 0.008 Hz and 0.09 Hz. CompCor (Behzadi et al., 2007; Chai et al., 2012) noise components within white matter and CSF were estimated by computing the average BOLD signal as well as the largest principal components orthogonal to the BOLD average, motion parameters, and outlier scans within each subject’s eroded segmentation masks. From the number of noise terms included in this denoising strategy, the effective degrees of freedom of the BOLD signal after denoising were estimated to range from 89.1 to 111.7 (average 104.9) across all subjects (Nieto-Castanon, 2022).

##### First-level analysis (SBC)

Seed-based connectivity maps (SBC) and ROI-to-ROI connectivity matrices (RRC) were estimated characterizing the patterns of functional connectivity with specific ROIs. Functional connectivity strength was represented by Fisher-transformed bivariate correlation coefficients from a weighted general linear model [weighted-GLM (Nieto-Castanon, 2020d)], defined separately for each pair of seed and target areas, modelling the association between their BOLD signal time-series. In order to compensate for possible transient magnetization effects at the beginning of each run, individual scans were weighted by a step function convolved with an SPM canonical hemodynamic response function and rectified.

Group-level analyses were performed using a General Linear Model [GLM (Nieto-Castanon, 2020d)]. For each individual voxel, a separate GLM was estimated, with first-level connectivity measures at this voxel as dependent variables (one independent sample per subject and one measurement per task or experimental condition, if applicable), and groups or other subject-level identifiers as independent variables. Voxel-level hypotheses were evaluated using multivariate parametric statistics with random effects across subjects and sample covariance estimation across multiple measurements. Inferences were performed at the level of individual clusters (groups of contiguous voxels). Cluster-level inferences were based on parametric statistics from Gaussian Random Field theory (Worsley et al., 1996; Nieto-Castanon, 2020a). Results were thresholded using a combination of a cluster-forming *p* < 0.001 voxel-level threshold, and a familywise corrected p-FWE < 0.05 cluster-size threshold. Additionally, for the RRC analysis, p-FWE correction at the ROI-level was applied to ensure control of false positives (Zalesky et al., 2010).

Seed-based and ROI-to-ROI functional connectivity analyses were performed using the default ROI sets implemented in the CONN toolbox. Network-level analyses were based on predefined ROIs representing canonical large-scale functional networks (e.g., frontoparietal and dorsal attention networks).

### Statistical analysis

The statistical analysis of the electrophysiological and neurocognitive data was performed using Jamovi (ver. 2.3.18). The normality of the data was assessed using the Kolmogrov-Smirnov test. The Wilcoxon signed-rank test was used to compare data. The Mann Whitney-U test was used for group comparisons between variables. The mean rank was provided for comparisons of demographic, cognitive, and behavioral information groups with non-normal distribution. Additionally, mean ± SD was given. Chi square analysis was used to examine categorical variables. We used repeated measure ANOVA to evaluate the neurocognitive score alterations in active tACS group compared to sham group following tACS intervention. The significance level for all comparisons was determined as *p* ≤ 0.05.

## Results

### Demographic features and baseline neurocognitive scores of the participants before tACS stimulation

All participants were randomly assigned to either the sham or the stimulation group. However, we still applied the Mann–Whitney U test to identify any demographic or baseline neurocognitive score differences before tACS between the groups. We did not observe any significant difference in years of education and major neurocognitive scores (such as MoCA, MMSE, working memory, executive function scores, HDRS) between groups. However, a significant difference was found in age (Table 1) which led us to add age and gender values as covariates for out further connectivity analysis.

**Table 1:** Demographic features and baseline neurocognitive scores of the participants before tACS stimulation.

Variables	Real stimulation (ITF-1) group (*n* = 10)		Sham stimulation group (*n* = 12)		*p* values
	Mean ± SD	Median (IQR)	Mean ± SD	Median (IQR)	
Age	29.00 ± 3.43	27.5 (6.5)	25.58 ± 3.73	24 (0.25)	0.004*
Years of education	18.00 ± 0.00	18 (0)	17.8 ± 0.45	18 (0.25)	0.108
Digit span forward	6.50 ± 1.35	7 (2.75)	6.17 ± 0.83	6 (0.25)	0.487
Digit span backward	4.50 ± 1.84	5 (1)	4.50 ± 0.91	5 (1)	0.700
WMS logical memory	17.40 ± 2.50	17.5 (3.5)	14.17 ± 3.24	14.5 (4.25)	0.018*
WMS logical memory (long)	14.70 ± 5.01	15 (6.5)	13.58 ± 2.81	13.5 (3.75)	0.517
MMSE	29.50 ± 0.71	30 (1)	29.75 ± 0.45	30 (0.25)	0.418
MoCA	28.80 ± 1.32	29 (0.75)	28.42 ± 1.78	29 (3)	0.863
Immediate memory	7.30 ± 2.79	7 (1.75)	6.08 ± 2.07	6 (3.25)	0.254
Learning score	124.10 ± 15.26	128 (14.5)	122.92 ± 15.02	126.5 (15.8)	0.857
Total recall	14.90 ± 0.32	15 (0)	14.67 ± 0.65	15 (0.25)	0.377
Category fluency	18.80 ± 5.73	19.5 (6.75)	16.92 ± 4.25	15 (7.5)	0.387
Verbal fluency	35.90 ± 15.59	26.5 (19.8)	39.67 ± 12.24	37 (11)	0.428
Stroop 1	36.00 ± 5.58	35 (7.75)	29.25 ± 5.83	28 (2.25)	0.012*
Stroop 2	27.80 ± 5.25	26.5 (4.5)	24.08 ± 3.55	24 (0.5)	0.041*
Stroop 3	62.60 ± 18.04	60.5 (14.5)	51.75 ± 14.19	48 (18.8)	0.137
Trail making part A	33.35 ± 11.45	33 (16.1)	33.42 ± 8.99	33.5 (12.5)	0.988
Trail making part B	73.33 ± 27.27	69.5 (37.3)	69.17 ± 25.41	59.5 (20)	0.715
Naming	28.80 ± 3.05	30 (2.5)	29.17 ± 1.27	29 (2)	0.661
Benton	22.80 ± 1.99	23.5 (2.75)	23.58 ± 2.02	23.5 (2.25)	0.373
WMS visual	12.20 ± 2.04	12.5 (2.75)	13.25 ± 1.22	14 (1.25)	0.194
WMS visual long	12.50 ± 2.22	14 (3.25)	12.08 ± 2.61	13.5 (2.25)	0.548
HDRS	26.70 ± 3.74	27 (2.75)	27.33 ± 4.33	27 (5)	0.721

MMSE, Mini-Mental State Examination; MoCA, Montreal Cognitive Assessment; WMS, Wechsler Memory Scale; HDRS, Hamilton Depression Rating Scale. The asterisk (*) and bold values indicates that the *p* value is statistically significant (*p* < 0.05).

### Neurocognitive and electrophysiological alterations following tACS intervention

For the statistical analysis of the clinical data, Repeated Measures ANOVAs were performed with the 2-by-2 mixed design for each test. Time (pre-tACS, post-tACS) was the within-subjects factor and Group (real stimulation group, sham stimulation group) was the between-subjects factor in the design. A significant time-by-group interaction was observed for immediate memory scores (*F* = 4.61, *p* = 0.046). *Post hoc* pairwise comparisons revealed a significant improvement in immediate memory following tACS in the real stimulation group (*t* = 3.489, *p* = 0.013), an effect not observed in the sham group (Table 2). Additionally, we found a significant time-by-group interaction for verbal fluency score (*F* = 5.08, *p* = 0.037) with *Post Hoc* comparisons indicating a significant increase in post-tACS compared to pre-tACS in real stimulation group (*t* = 4.845, *p* < 0.001). Similarly, category fluency scores also showed a significant time-by-group interaction (*F* = 8.72, *p* = 0.008), where *post hoc* pairwise comparisons showed a significant increase in post-tACS compared to pre-tACS in real stimulation (Table 2). None of these findings were present in the sham group. We added age and gender as covariates to adjust potential confounding effects.

**Table 2:** Neurocognitive and electrophysiological alterations following tACS intervention.

Tests	Model’s p	*Post hoc* comparisons – Time*group				
		Group	Time	Mean difference	t	P_Tukey_
Immediate memory	**0.046**	Real	Post – Pre	4.106	3.489	0.013
		Sham	Post – Pre	0.474	0.433	0.972
Verbal fluency	**0.037**	Real	Post – Pre	13.51	4.845	<0.001
		Sham	Post – Pre	4.48	1.727	0.339
Category fluency	**0.008**	Real	Post – Pre	6.158	3.975	0.004
		Sham	Post – Pre	−0.416	−0.288	0.991
Individual theta band resting power	0.060	Real	Post – Pre	−0.306	2.49	0.092
		Sham	Post – Pre	0.025	−0.22	0.996

This table shows the repeated measures ANOVA test results. We performed a 2-by-2 mixed design for each test. Time (pre-tACS, post-tACS) was the within-subjects factor and Group (real stimulation group, sham stimulation group) was the between-subjects factor in the design. The statistical significance of the whole model is determined as “Mode’s p” in the table. Bold values indicates that the *p* value is statistically significant (*p* < 0.05).

### Electrophysiological results

**Figure 2:**
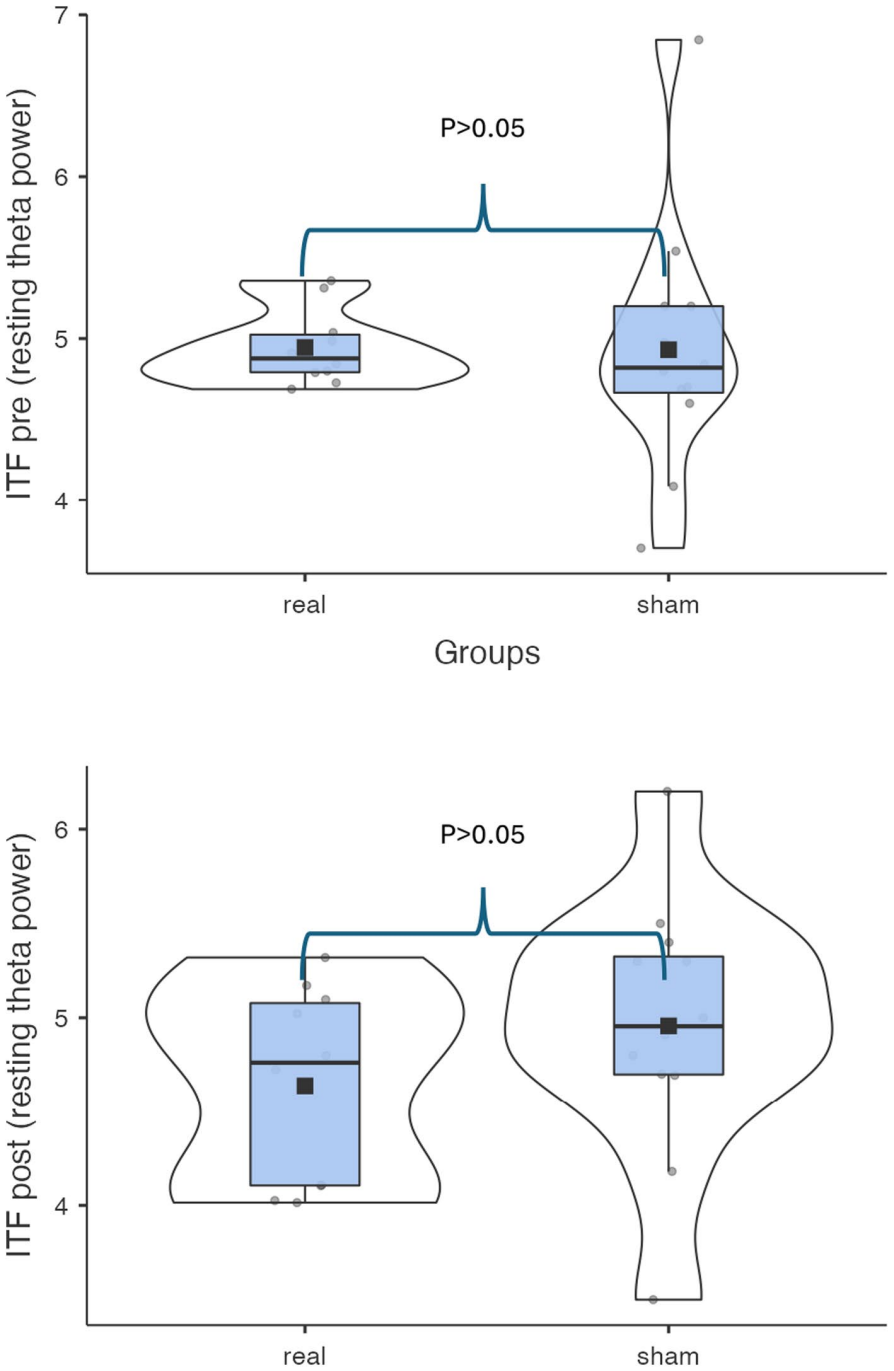
The vertical bars denote 0.95 confidence intervals. Dots represent the observed scores. ITF, individual theta frequency.

We found no significant differences in resting-state theta-band power values between the real and sham groups (*F* = 0.38, *p* = 0.544, and η^2^p = 0.01) (Figure 2). The ANOVA also revealed that resting-state theta-band power values decreased from pre-tACS to post-tACS (main effect of ‘time’: *F* = 2.84, *p* = 0.107, and η^2^p = 0.124), which was not depended on group (time*group interaction: *F* = 3.98, *p* = 0.06, and η^2^p = 0.166). Please note that although the time*group interaction not achieving statistical significance (*p* = 0.06), the Real ITF group exhibited a reduction in theta power after tACS while there were no tACS effects on the sham group (Table 2).

### Between-group (real group vs. sham group) differences of functional connectivity of dorsal attention and Frontoparietal networks between other parts of the brain

**Figure 3:**
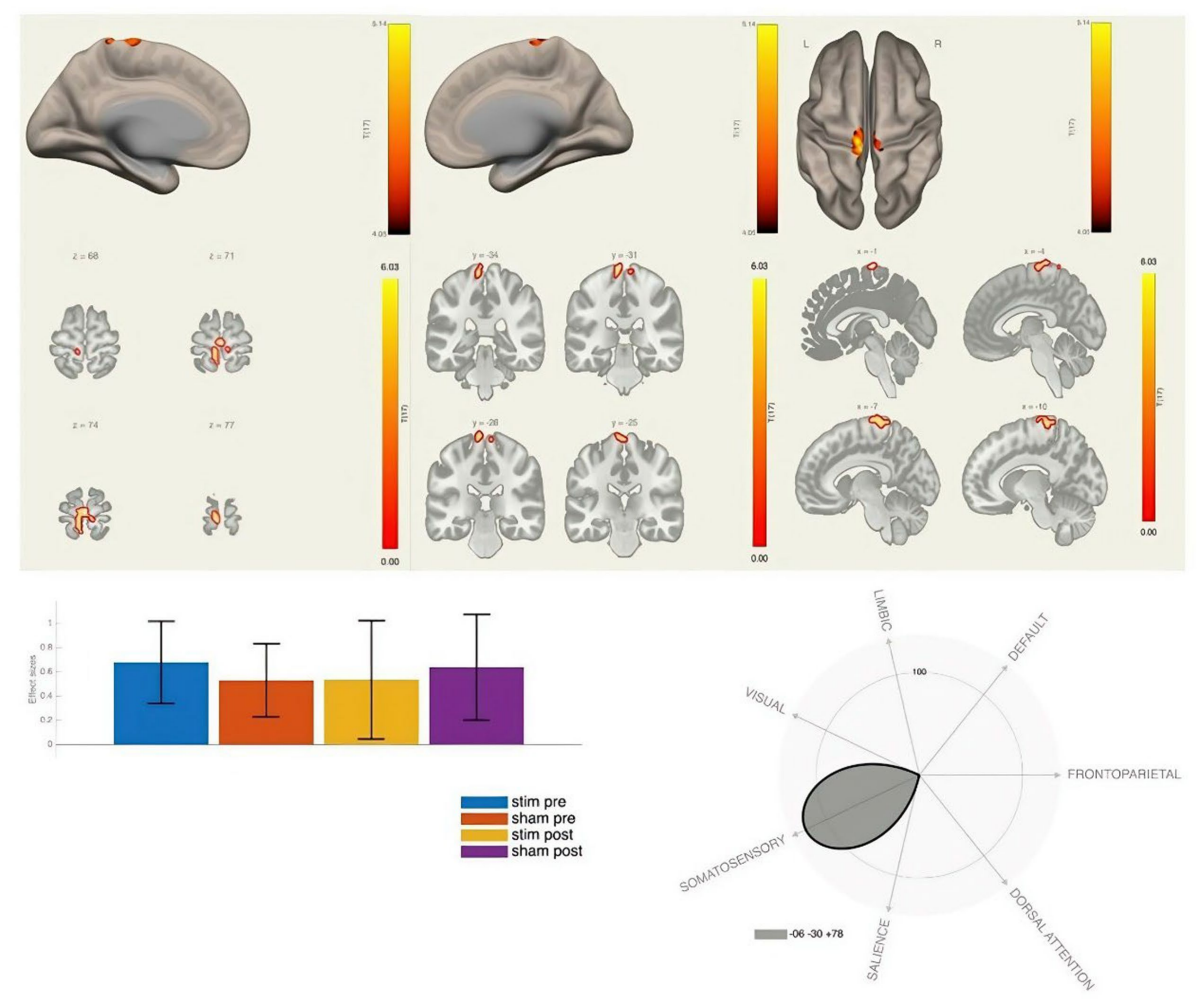
Between-group differences of functional connectivity between left frontal eye fields of dorsal attention networks with bilateral postcentral and precentral gyri. Surface and volumetric brain renderings (top panel) illustrate significant between-group differences in functional connectivity between the left frontal eye field (FEF) of dorsal attention network (DAN) and bilateral precentral and postcentral gyrus. Clusters exhibiting decreased connectivity in the real stimulation group compared to the sham group after intervention are displayed in warm color scales. The lower left bar plot depicts mean connectivity values between the left FEF of DAN and bilateral precentral and postcentral gyri across four conditions (stim-pre, stim-post, sham-pre, sham-post), with error bars indicating standard deviations. The lower right radar plot visualizes the network-level specificity of the observed connectivity changes, showing increased functional connectivity between left FEF of DAN and somatosensory regions (bilateral precentral and postcentral gyri) in sham group compared to real stimulation group.

**Figure 4:**
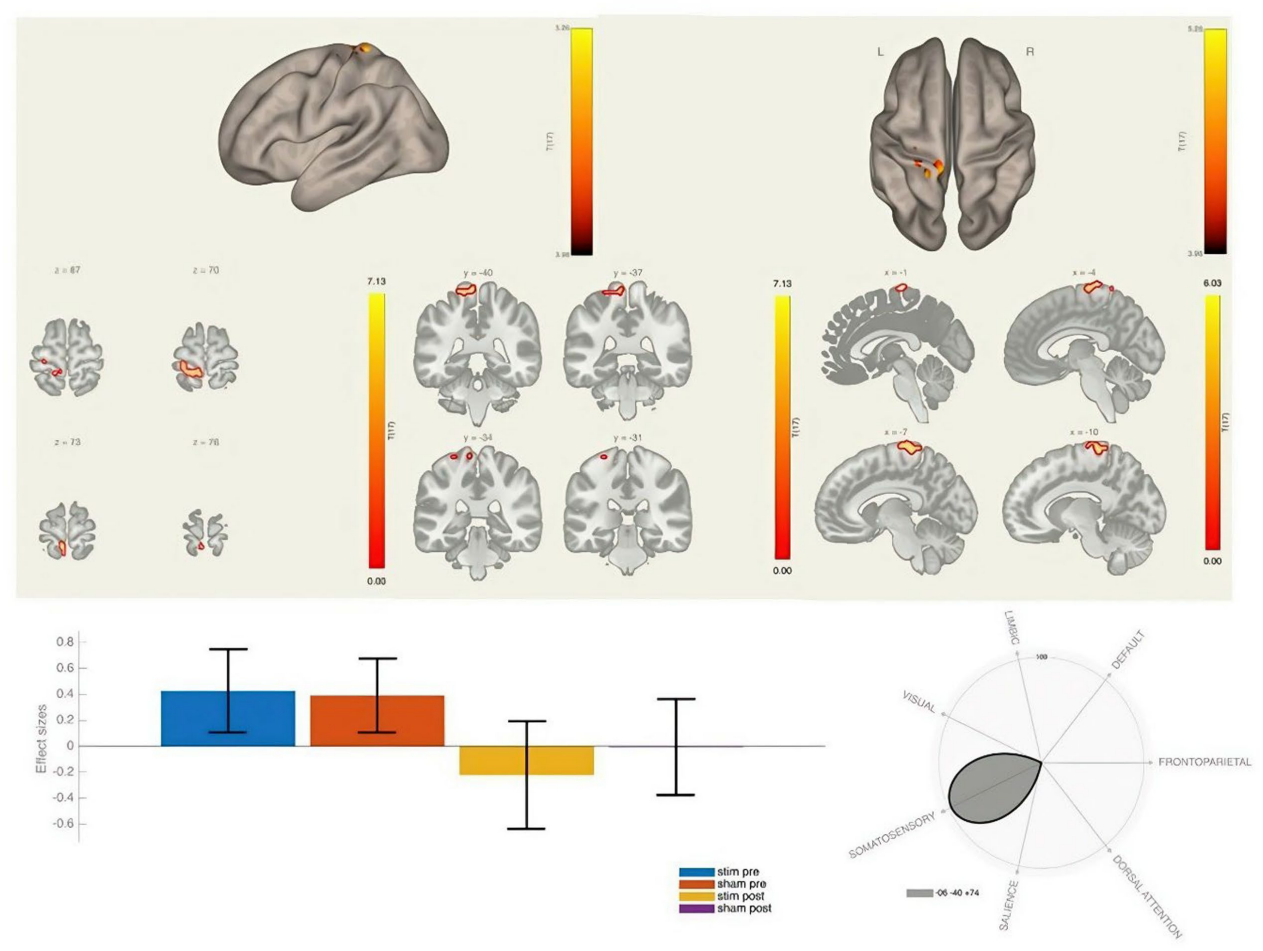
Between-group differences of functional connectivity between right frontal eye fields of dorsal attention networks with left postcentral and precentral gyri. This figure illustrates between-group differences in resting-state functional connectivity between the right frontal eye field (FEF) of dorsal attention network (DAN) and the left precentral and postcentral gyri following real and sham stimulation. The top panel presents cortical surface and volumetric renderings of statistically significant clusters showing altered connectivity. Warm color scales indicate regions where group-level effects emerged, with MNI coordinates labeled on axial, coronal, and sagittal slices. Higher *t* values reflect stronger between-group differences. The lower left panel shows group-wise bar plots of standardized effect sizes (*β* coefficients) across four conditions: pre real stimulation (blue), pre sham stimulation (orange), post real stimulation (yellow), and post sham stimulation (purple). Notably, connectivity values in the real stimulation group (yellow bar) appear decreased following intervention.

**Figure 5:**
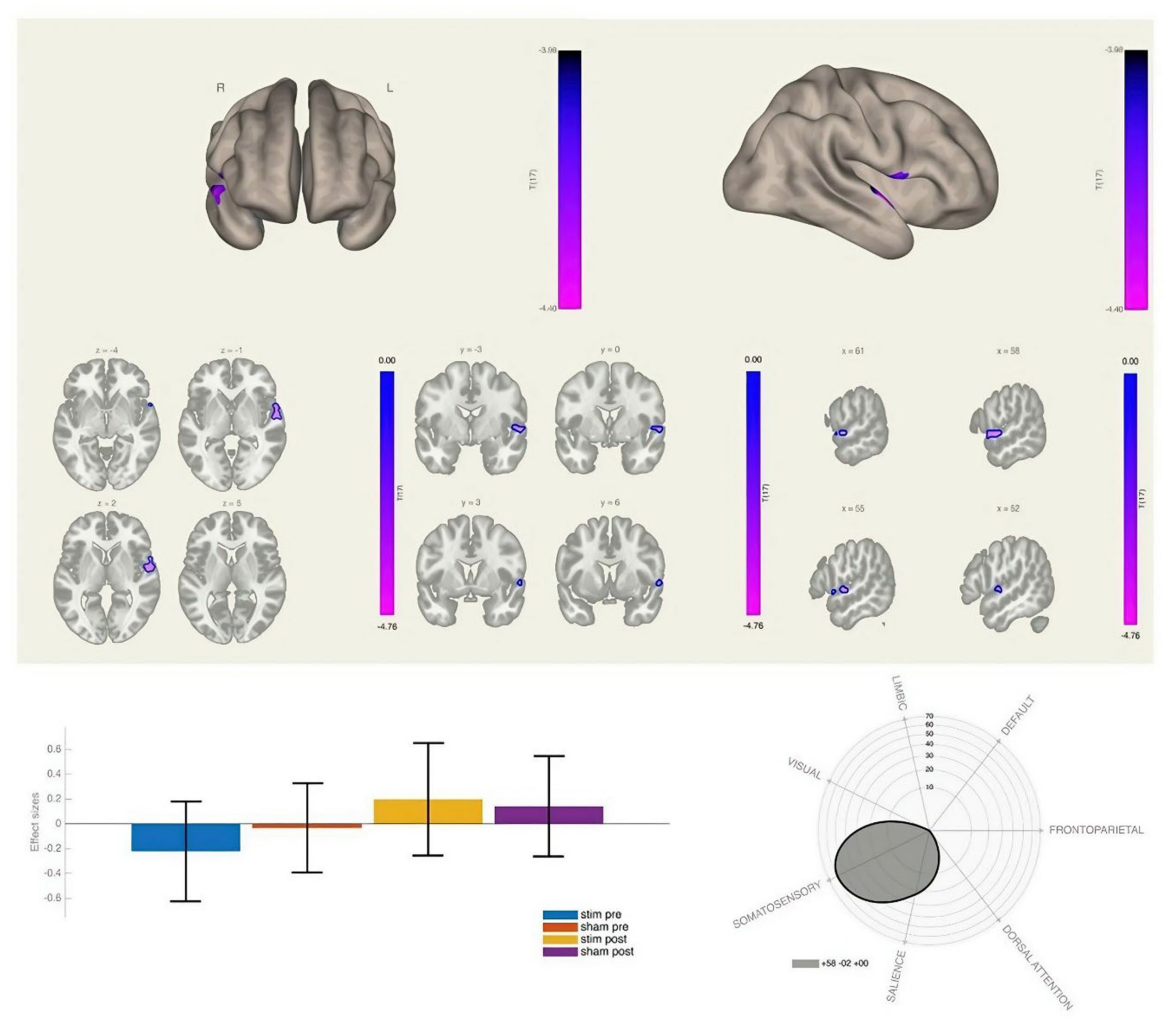
Between-group differences of functional connectivity between left lateral prefrontal cortex of frontoparietal network with right planum polare and central opercular cortex. This figure presents between-group differences in resting-state functional connectivity between the left lateral prefrontal cortex (lLPFC) of the frontoparietal network (FPN) and the right planum polare and central opercular cortex following stimulation. The top panel displays cortical surface and volumetric brain maps highlighting regions where significant connectivity differences were observed. Color maps use a cool scale (violet to blue) to indicate negative *t*-values (Sham>Stim), reflecting decreased connectivity in sham group compared to real stimulation group following intervention (*p* < 0.005, uncorrected). Brain slices are shown in MNI coordinate space across axial, coronal, and sagittal planes. The lower left bar plot presents group-level effect sizes (standardized β coefficients) for the four conditions: real stimulation pre (blue), sham pre (orange), real stimulation post (yellow), and sham post (purple).

In determining the Dorsal Attention and Frontoparietal networks as seed Roi, we have found significantly decreased connectivity of dorsal attention network with bilateral frontal eye fields as compared to before the tACS intervention in the real group (*p* < 0.05) (Table 3; Figures 3, 4). We also determined significantly increased connectivity of Frontoparietal network with left lateral prefrontal cortex as compared to before the tACS intervention in the real group (p < 0.05) (Table 3; Figure 5).

**Table 3:** Between-group (real group vs. sham group) differences of functional connectivity of dorsal attention and frontoparietal networks between other parts of the brain.

Network sham>stim	Seed (node)	Target cluster	MNI coordinates	size	t-max	p-FWE	p-FDR
Dorsal attention network	Left frontal eye fields	Bilateral precentral gyrus	−06, −30, 78	188	5.27	0.000281	0.00024
		Bilateral postcentral gyrus					
	Right frontal eye fields	Left postcentral gyrus	−06, −40, 74	109	8.38	0.015059	0.0169
		Left precentral gyrus					
Frontoparietal network	Left lateral prefrontal cortex	Planum polare right	+58, –02, +00	82	−4.38	0.0578	0.0302
		Central opercular cortex					

For each seed region (node) within the Dorsal Attention Network and Frontoparietal Network, significant clusters are reported. MNI coordinates correspond to the peak voxel (maximum *t* value) within each cluster. Cluster size indicates the number of voxels within the significant cluster. Statistical significance was assessed at the cluster level using FWE- and FDR-corrected *p* values. Cluster locations may include multiple anatomical regions reflecting the spatial extent of each cluster.

### Between-group interaction effects of network connectivity and verbal fluency in dorsal attention and Frontoparietal networks (real vs. sham)

**Figure 6:**
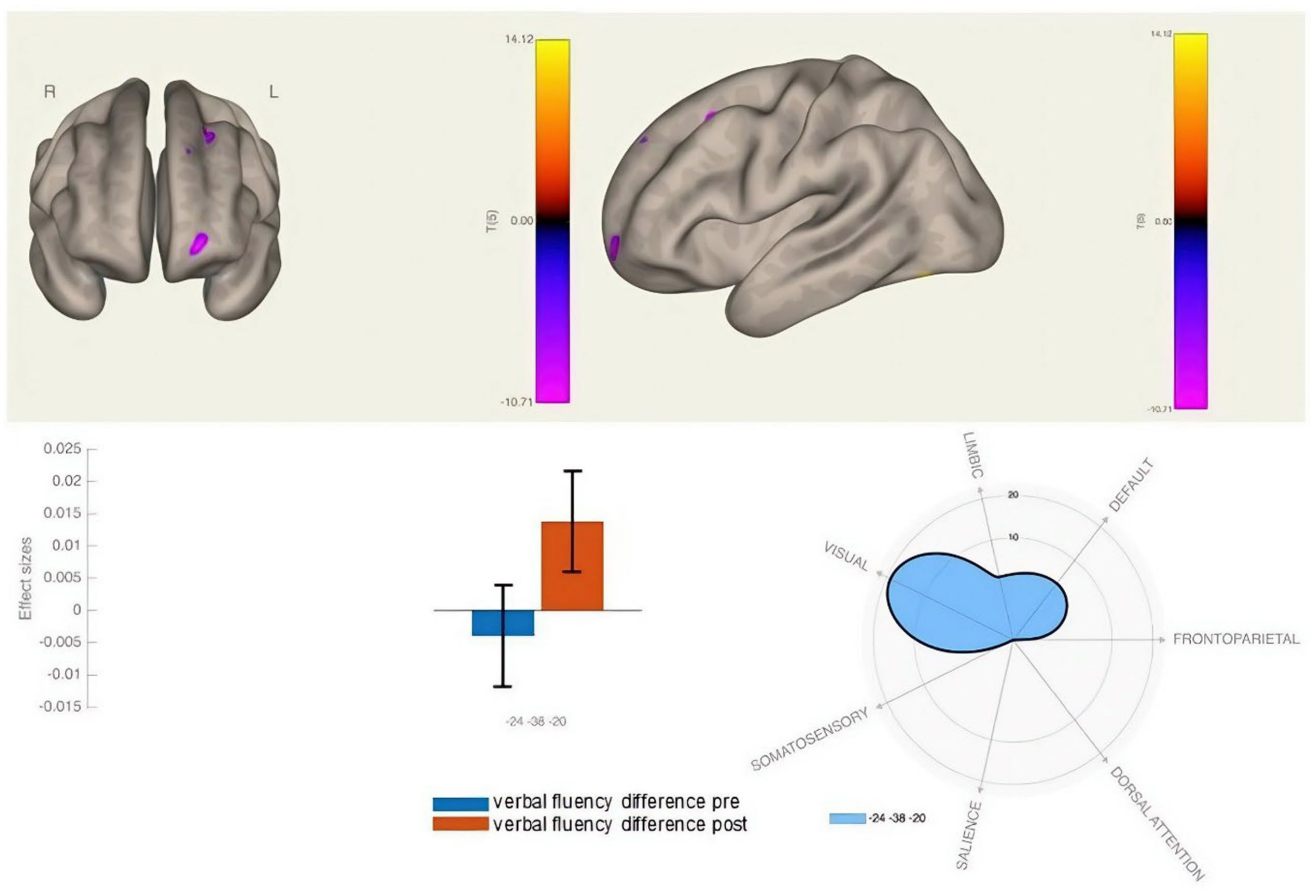
The connectivity of dorsal attention network with posterior division of left temporal fusiform cortex was significantly interacted with verbal fluency scores in the Stim group.

**Figure 7:**
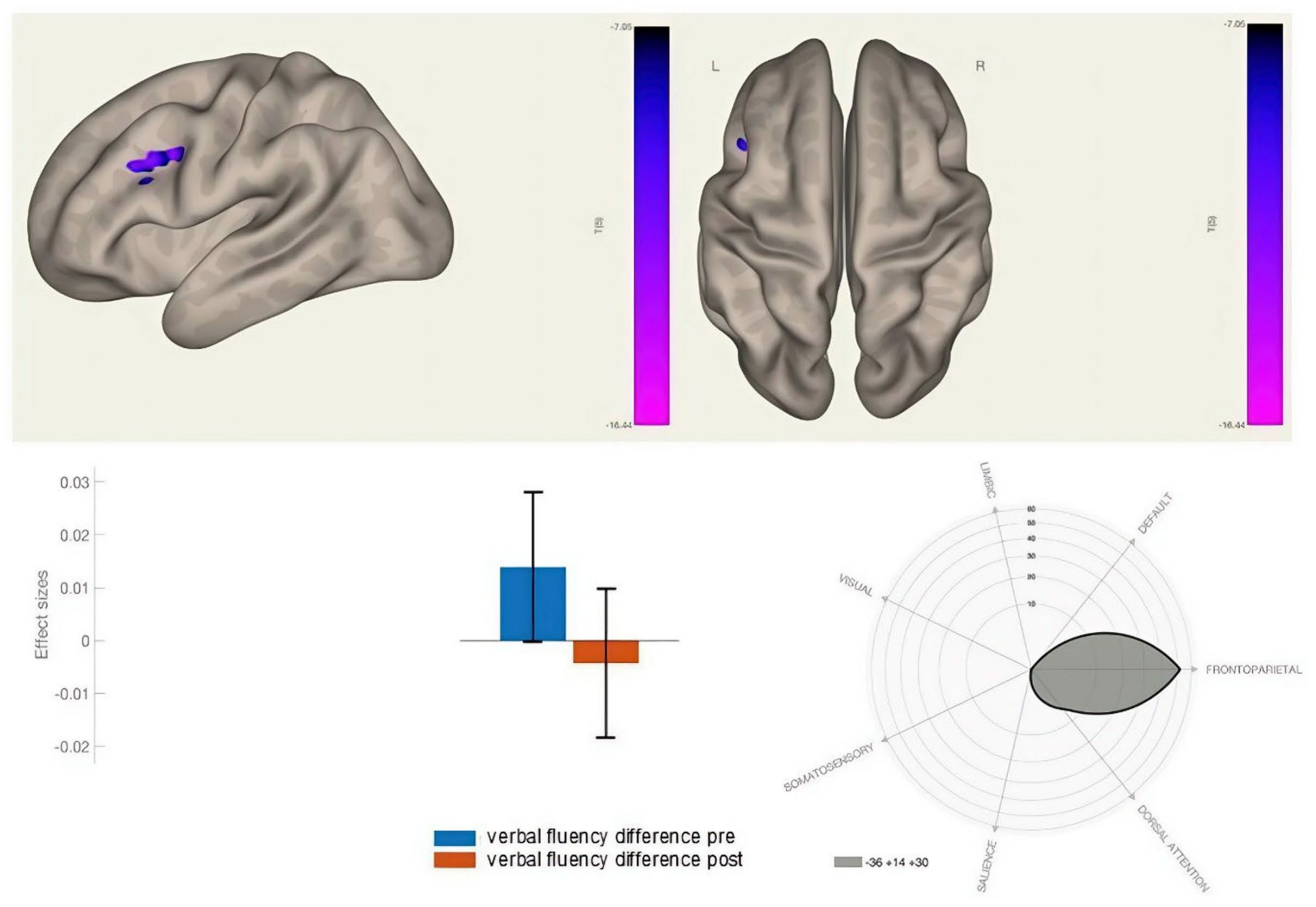
Frontoparietal network’s connectivity with left middle frontal gyrus was significantly interacted with the verbal fluency scores.

Among the functionally significant regions, the connectivity of dorsal attention network with posterior division of left temporal fusiform cortex was significantly interacted with verbal fluency scores in the Stim group (p-FDR = 0.014377) (Table 4 and Figure 6). Also, frontoparietal network’s connectivity with left middle frontal gyrus was significantly interacted with the verbal fluency scores (p-FDR: 0.000017) (Table 4 and Figure 7). We added age, gender and years of education as covariates to all models for adjusting their effects.

**Table 4:** Regression analysis showed significant interactions between verbal fluency scores and functional connectivity of dorsal attention network with posterior division of left temporal fusiform cortex and frontoparietal with left middle frontal gyrus.

Seeds	Cluster locations	Cluster coordinates	Size	t-max	p-FDR	p-FWE	P-uncorr
Positive correlation with verbal fluency scores							
Dorsal attention network	Posterior division of left temporal fusiform cortex	−24, −38, −20	28	7.43	0.01437	0.0297	*p* < 0.001
Negative correlation with verbal fluency							
Frontoparietal network	Left middle frontal gyrus	−36, +14, + 30	81	−22.03	0.000017	*p* < 0.001	*p* < 0.001

## Discussion

The present study investigated the cognitive and neurophysiological after-effects of personalized EEG-based theta-frequency tACS, which was configured to either entrain or attenuate endogenous theta oscillations with the objective of enhancing memory capacity. Specifically, we aimed to experimentally attenuate theta oscillations by administering tACS stimulation at one Hertz below the individual peak theta frequency (ITF-1; attenuation) and to compare clinical and fMRI outcomes of real stimulation with sham stimulation.

Several electrophysiological studies have shown that slower-frequency theta oscillations may facilitate enhanced memory performance by accommodating additional gamma-range activity at a preferred theta phase, defined as a type of theta-gamma cross-frequency coupling (associated with enhanced cognitive functions) (Polanía et al., 2012; Jaušovec and Jaušovec, 2014; Wolinski et al., 2018; Sreeraj et al., 2019). For instance, several studies have demonstrated that tACS applied to alter the frequency of oscillations may influence cognitive functions such as working memory and perception (Jaušovec and Jaušovec, 2014; Sreeraj et al., 2019). The most consistent outcome across previous EEG studies on tACS after-effects has been an increase in resting-state EEG amplitude accompanied by heightened fronto-central power associated with improved cognitive outcome. For instance, Aktürk et al. (2022) recently discovered increased fronto-central power solely in ITF-specific conditions. Conversely, Polanía et al. (2012) identified negative effects on working memory performance following anti-phase fronto-parietal theta tACS. However, their experiment concentrated on online entrainment rather than after-effects related to synaptic plasticity, as observed in the current experiment.

In contrast to the above studies focusing on temporal resolution, we adopted a spatial approach with a dynamic neuroimaging method. For the first time in the literature, our evaluation dynamically examined whether cognition is enhanced following the application of tACS and whether these effects are mediated by neurophysiological/neuroimaging markers that can predict the tACS-induced enhancements in cognitive function in healthy individuals. We applied tACS during a resting state and examined the cognitive effects through fMRI measurements before and after tACS administration with a comprehensive battery of behavioral assessments to evaluate detailed attention, learning, and memory performance. The overall results can be summarized across the main domains of cognition and brain connectivity changes as shown below.

Our cognitive results are consistent with the majority of prior theta tACS studies, showing a beneficial after-effect of theta tACS on cognitive performance metrics (Polanía et al., 2012; Jaušovec and Jaušovec, 2014; Wolinski et al., 2018; Sreeraj et al., 2019). Furthermore, our study demonstrated that individualized theta tACS (ITF − 1 Hz) may produce beneficial after-effects on cognitive performance measures; however, it does not clarify whether these effects differ from those achieved with non-individualized theta tACS. To address this question, future studies could include an additional non-individualized theta tACS group, allowing a direct comparison between individualized and standard stimulation protocols. We further identified advantageous effects on learning scores during a neuropsychological battery applied well after the completion of tACS. Importantly, the memory and fluency (both category and verbal) cognitive subdomains seemed to be susceptible to the tACS effect compared to other subdomains. This is in line with theoretical models positing that memory or executive capacity can be augmented via extended theta cycles (lower theta frequency) confirmed with selective memory enhancements within the ITF-1 Hz group (Jaušovec and Jaušovec, 2014).

In this respect, other memory tasks and neuropsychological assessments (Trail making part B, STROOP test, MoCA and MMSE scores and Wechsler memory scale scores) revealed no significant tACS effects. As mentioned above we applied individual-specific (individual’s theta frequency minus 1 Hz) tACS stimulation and demonstrated prominent cognitive effects, a finding which aligns with the previous literature. A good example of this is Sreeraj et al.’s in which ‘online tACS’ was applied to schizophrenia patients. Those administered tACS while the patient was engaged in a task and found that working memory and attention functions improved following the stimulation, which is also in line with our results (Sreeraj et al., 2019). Additionally, there are also studies showing theta-tACS improving working memory functions in healthy subjects (Jaušovec and Jaušovec, 2014). To summarize, we observed tACS-induced enhancements in short-term memory as well as category fluency and verbal fluency test scores in the real stimulation group.

In unique contrast to the previous tACS memory literature, our investigation employed EEG measurements specifically designed to slow an individually tailored theta frequency. This methodological distinction is of critical importance, since the most prominent cognitive after-effects were confirmed exclusively in the real (Individual Theta Frequency minus 1 Hz [ITF-1]) stimulation group compared to the sham group. Our fMRI analysis revealed significantly altered activity in the dorsal attention network (DAN) and frontoparietal network (FPN) in the REAL tACS group after tACS stimulation compared to SHAM group. As explicitly hypothesized, this was anticipated since tACS has previously been confirmed to exert a pronounced effect on FPN and dorsal attention networks (Draaisma et al., 2022; Kemmerer et al., 2022; Pupíková et al., 2024; Jouzdani et al., 2025). Our findings of significantly changed dorsal attention and frontoparietal network activity associated with improved verbal fluency, confirmed by our detailed regression analysis-prominent only in the stimulation group (Figures 6, 7) also align with a recent study by Aktürk et al. (2022) showing significantly altered frontoparietal EEG activity confirmed through EEG power spectrum analysis. Although we did not evaluate direct electrophysiological evidence for these networks, due to the study concept, following ITF-1 tACS, changes in dorsal attention and frontoparietal network connectivity appear to represent a potential neural mechanism underlying lasting behavioral improvements induced by slowing theta frequency to an optimal level (Reinhart and Nguyen, 2019). This aligns with recent research by Reinhart and Nguyen (2019), who also suggested that memory enhancements from tACS may result from neuroplastic changes in functional connectivity via theta-gamma modulation. This could represent a broader principle in which EEG-informed, individually tailored frontoparietal tACS can promote sustained cognitive improvements associated with critical fMRI changes by stimulating slightly below (or above, depending on network demands) an individual’s peak frequency.

As explicitly hypothesized, these findings are what we expected since tACS has consistently been shown to have the most pronounced effect on FPN and dorsal attention networks, as previously confirmed through EEG power spectrum analysis studies (Draaisma et al., 2022; Kemmerer et al., 2022; Pupíková et al., 2024; Jouzdani et al., 2025).

Although our findings suggest that personalized left frontoparietal tACS may enhance memory associated with relevant fMRI connectivity changes, some limitations must also be acknowledged.

First, in terms of methodology, we determined ITF based on a global (whole head) EEG power spectrum, since the current literature contains no clear consensus or optimal means to estimate ITF (Jaušovec and Jaušovec, 2014; Reinhart and Nguyen, 2019). Some researchers have defined ITF using alpha frequencies, while others have relied on measures such as pre-stimulus coupling to gamma activity or phase-locking synchronization between specific brain regions during memory tasks. However, this requires a precise approach that might involve estimating ITF solely from task-relevant areas. Second, we did not include a theta-control group, and no significant difference in resting theta power was detected, despite a decrease in the real group and a slight increase in the sham group. This may be attributable to the resting design of this study, which aligns with the unchanged resting iTF scores after tACS application reported by Aktürk et al. (2022), despite a significant difference being observed in event-related iTF scores in their study. Nevertheless, it is difficult to conclude that theta slowing is effective in the absence of an additional theta control group. Another limitation of this study is the potential ceiling effect in behavioral tests, since our participants, healthy, well-educated young adults, scored near the maximum, which may have masked detectable improvements. It may therefore have been difficult to detect actual tACS-related score changes in many of the tests. However, this primarily affects null findings rather than positive effects. That in turn represents a limitation of our null findings, and not our positive results. Another potential limitation of the present study is the use of the conventional 10–20 EEG system (F3) for targeting the dorsolateral prefrontal cortex (DLPFC), rather than MRI-guided neuronavigation. Previous studies have demonstrated that electrode placement based on the 10–20 system may not precisely correspond to the intended cortical target due to inter-individual anatomical variability (De Witte et al., 2018). Additionally, data on the sensations experienced by participants in each group were not collected to assess whether blinding was properly implemented, which may represent an important limitation of the study. Additionally, our study had a relatively small sample size, and larger-scale studies with greater sample sizes are needed in future research.

Overall, our results provide new insights into connectivity alterations underlying tACS-induced behavioral enhancements that outlast stimulation. These may open the door to real-world applications of using non-invasive brain stimulation in cognitive rehabilitation and/or neurocognitive enhancement. Reliable after-effects of tACS would substantially broaden the range of applications of this technology. Despite these limitations, we believe that our results provide new insights into connectivity alterations underlying tACS-induced cognitive enhancements that outlast stimulation. We intend to adopt our different methodological approach as both a limitation and motivation in future task-specific functional magnetic resonance imaging (fMRI) studies.

In conclusion, tACS, a well-established non-invasive brain stimulation technique, has demonstrated cognitive benefits in various conditions. This study reinforces the existing literature, indicating notable enhancements in short-term memory and fluency (both verbal and category) after 5 days of tACS stimulation. Further investigations involving larger participant groups are now needed to consolidate and expand upon these findings. Although there is an extensive range of behavioral and fMRI measures that were incorporated in this study, the findings are encouraging but require replication.

## Data Availability

The raw data supporting the conclusions of this article will be made available by the authors, without undue reservation.
